# Reconstruction of the Articular Surface in the Subtalar Joint by Osteochondral Autologous Transplantation After Failure of Tibiotalocalcaneal Fusion With a Retrograde Nail: A Case Report

**DOI:** 10.7759/cureus.45654

**Published:** 2023-09-20

**Authors:** Junichi Sumii, Tomoyuki Nakasa, Yasunari Ikuta, Akinori Nekomoto, Nobuo Adachi

**Affiliations:** 1 Department of Orthopaedic Surgery, Graduate School of Biomedical and Health Sciences, Hiroshima University, Hiroshima, JPN; 2 Department of Artificial Joints and Biomaterials, Graduate School of Biomedical and Health Sciences, Hiroshima University, Hiroshima, JPN

**Keywords:** tibiotalar joint, subtalar joint, nonunion, osteochondral autologous transplantation, intramedullary nail, tibiotalocalcaneal arthrodesis

## Abstract

Tibiotalocalcaneal arthrodesis (TTCA) using the intramedullary nail has been conducted for severe deformity of both ankle and subtalar joints. While good clinical outcomes have been reported for TTCA, its nonunion rate is relatively high. We report a case of a 65-year-old male with nonunion of the tibiotalar joint and destruction of the subtalar joint after TTCA using a retrograde intramedullary nail. For this patient, we conducted a salvage procedure for the subtalar joint along with revision surgery for the tibiotalar joint to achieve bone union. The intramedullary nail was removed and the tibiotalar joint was debrided. Two osteochondral plugs were harvested from the lateral aspect of the talus and transplanted to the subtalar joint. The tibiotalar joint was fixed using screws and staples, with bone grafting. Magnetic resonance imaging (MRI) at six months after surgery showed that the articular surface of the subtalar joint was flushed and the osteochondral plugs were united with the surrounding bone. At one year and three months after surgery, the pain in the tibiotalar and subtalar joints had completely disappeared. Plain radiographs revealed that bone union of the tibiotalar joint and joint space of the subtalar joint was maintained. Japanese Society for Surgery of the Foot (JSSF) hindfoot scale improved from 53 points to 84 points at the final follow-up. Reconstruction of the subtalar joint using osteochondral autologous transplantation is a useful technique for failure cases with nonunion of the tibiotalar and subtalar joints after TTCA.

## Introduction

For severe osteoarthritis (OA) of the ankle, arthrodesis for the ankle joint is frequently performed to relieve pain and obtain stability [1]. Despite the loss of ankle joint motion, activities of daily living improved with ankle arthrodesis from the preoperative level, with high satisfaction of patients. While ankle arthrodesis could not restore normal gait parameters, cadence, velocity, and stride length were reportedly improved in the gait analysis [2]. After ankle arthrodesis, the Chopart and subtalar joint mobility increased to compensate for a stiff ankle, which may contribute to the patient’s satisfaction.

Tibiotalocalcaneal arthrodesis (TTCA) with a retrograde intramedullary nail has been used to treat severe deformities in both the ankle and subtalar joint due to OA or rheumatoid arthritis [3]. While good clinical outcomes have been obtained by TTCA, a high complication rate has also been described [4,5]. In particular, the fusion rate varied from 52% to 96.6% [6]. The necessity of subtalar joint cartilage resection is considered for TCCA with a retrograde intramedullary nail. Mulhern et al. suggested that eliminating cartilage resection at the subtalar joint might negatively influence patient outcomes in the TTCA [7]. However, remarkable subtalar joint deterioration was observed despite a very short follow-up period, suggesting that skipping the cartilage resection in the subtalar joint might have a negative impact on this structure [8]. In these cases of nonunion, the intramedullary nail should be removed during revision surgery, and large defects in the articular surface of the subtalar joint need to be addressed. We encountered a case with nonunion of the ankle joint and destruction of the subtalar joint after TTCA using the retrograde intramedullary nail. For this patient, we performed a salvage procedure for the subtalar joint along with revision surgery for the ankle joint to obtain the bone union.

## Case presentation

**Figure 1:**
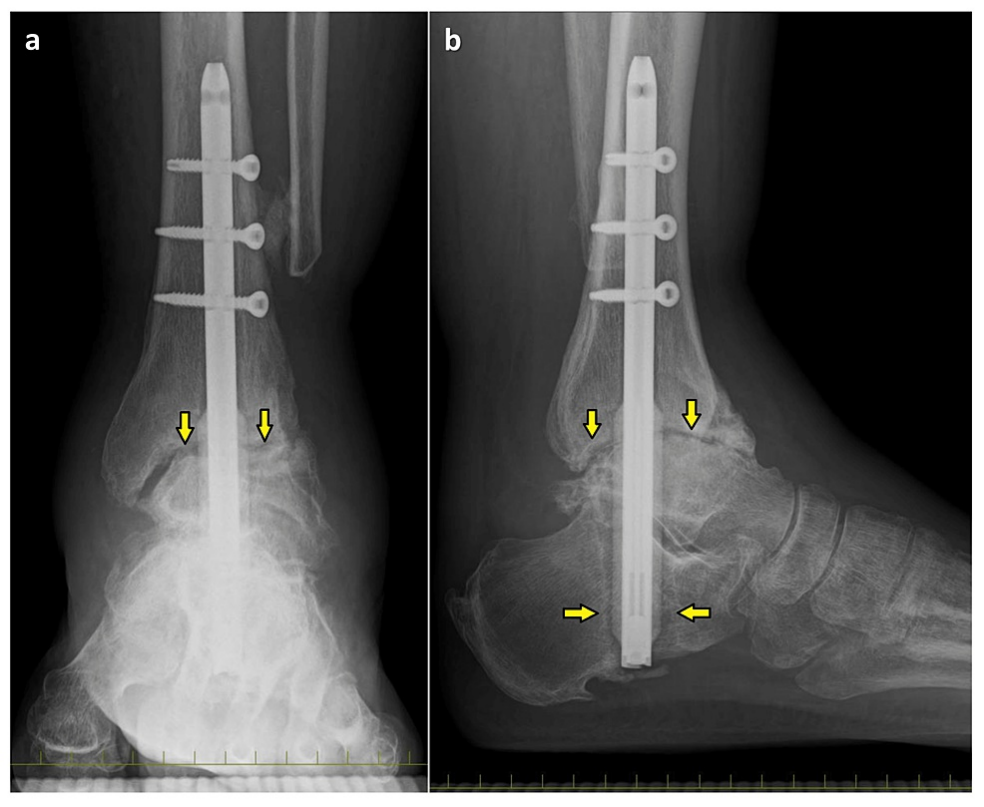
Preoperative plain radiographs at standing position (a) anteroposterior and (b) lateral images

A 65-year-old man had undergone ankle arthrodesis for OA of the ankle using the retrograde intramedullary nail eight months ago. However, he complained of severe pain at the tibiotalar and subtalar joints and plantar sensory disturbance. Therefore, he was referred to our hospital for further treatment. His left ankle exhibited swelling and tenderness in the tibiotalar and subtalar joints. The range of motion of the Chopart joint was 0° in dorsiflexion and 5° in plantar flexion, accompanied by pain. The range of motion in supination and pronation of the hindfoot was 5°, accompanied by pain. On plain radiographs, bone union was not achieved in the ankle joint, with an enlargement of the intramedullary nail canal in the talus and calcaneus, which was more pronounced distally (Figure 1).

**Figure 2:**
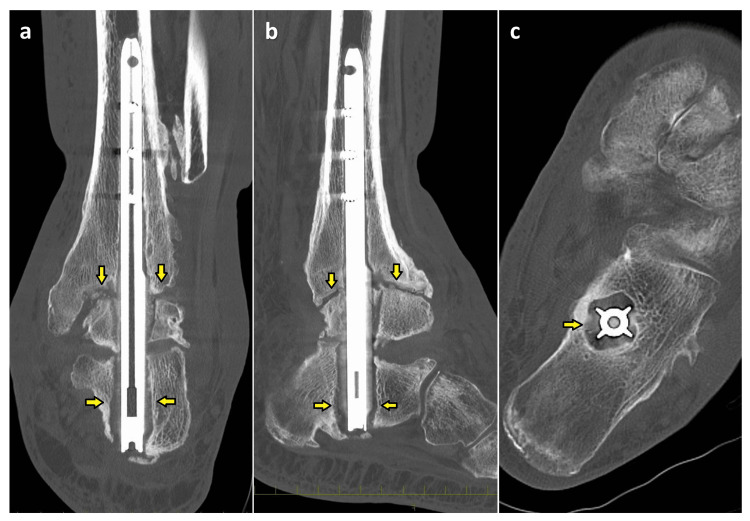
Preoperative CT (a) coronal, (b) sagittal, and (c) axial images CT, computed tomography

The lateral malleolus was removed 10 cm from the medial malleolus tip. The intramedullary nail was transfixed with three screws at the tibia. The distal part of the intramedullary nail was not fixed to the calcaneus, because an intramedullary nail with fin-like longitudinal ridges was used. On computed tomography (CT), nonunion was observed in the ankle joint and the intramedullary nail canal was enlarged, particularly in the calcaneus (Figure 2).

**Figure 3:**
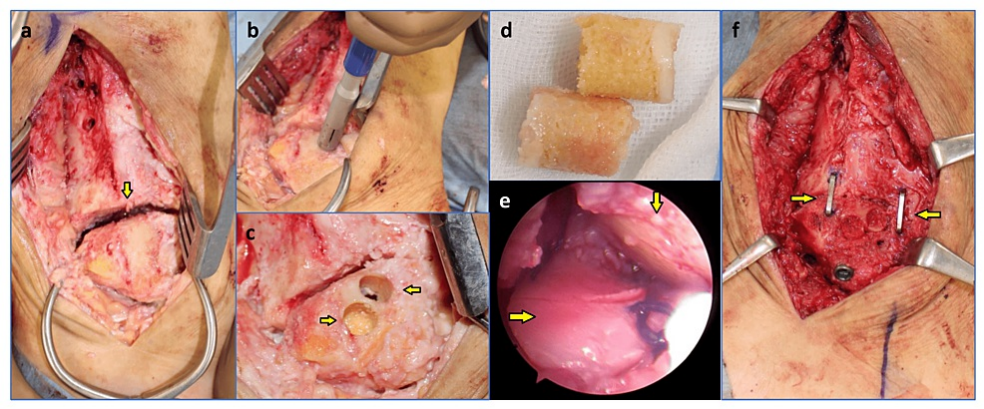
Operative findings (a) After the removal of the intramedullary nail and curettage of the tibiotalar joint. (b) The harvesting of the osteochondral plugs. (c) After the harvest of osteochondral plugs from the lateral aspect of the talus. (d) Harvested osteochondral plugs. (e) Arthroscopic findings of the subtalar joint after the osteochondral autologous transplantation. (f) After the fixation of the tibiotalar joint.

The joint contour in the subtalar joint was intact, except for the intramedullary nail canal. which led to the idea that preservation of the subtalar joint by the reconstruction of the cartilage surface would provide a better clinical outcome than TTCA because of the mobility of the subtalar joint. For ankle arthrodesis, a combination of screws and compression staples would be planned to use because this combination can provide firm fixation [9]. Because his symptoms did not improve, surgery to unite the ankle joint and reconstruct the articular surface of the subtalar joints was performed. Because of the presence of intact articular cartilage in the subtalar joint except for the enlarged intramedullary nail canal, we chose osteochondral autologous transplantation to reconstruct the articular surface of the subtalar joint. In our case, the osteochondral plugs were harvested from the lateral aspect of the talus. The patient was placed in the lateral position, and his tibia, talus, and calcaneus were exposed via an initial skin incision of approximately 10 cm on the lateral aspect of the ankle. Three screws for the nail at the tibia were exposed and removed. Then, the intramedullary nail was removed through the initial plantar skin incision. The ankle joint was filled with scar tissue and mobilized. All sclerotic or necrotic bones in the bony surface of the talus and tibia were debrided to prepare for ankle fusion (Figure 3a). The lateral malleolus was resected at the initial surgery, and articular cartilage on the lateral aspect of the talus remained. As the remained articular cartilage of the talus appeared to be normal, two cylindrical osteochondral plugs with a 10-mm diameter were harvested using the osteochondral autograft transfer system (OATS; Arthrex Inc., Naples, FL, USA) (Figure 3b-Figure 3d). Two cylindrical bone plugs of the same size were harvested from the iliac crest along with the cancellous bone chip. The talus was inverted to expose the intramedullary canal through the ankle joint, and two osteochondral grafts were placed at the talus and calcaneus through the intramedullary canal with the cancellous bone chips as the articular surface of the osteochondral plugs. The osteochondral grafts were then transfixed using bioabsorbable pins with a diameter of 1.5 mm (Superfixorb, Depuy, Raynham, MA, USA). It was confirmed that the articular surface of the subtalar joint was flushed using a 2.7 mm arthroscope (Figure 3e). Subsequently, the ankle joint was fixed with two cannulated cancellous screws with two 6.5 mm diameters (Ace Medical, El Segundo, CA, USA), and two compression staples (DynaNite; Arthrex, Inc., Naples, FL, USA) with a cancellous bone chip, and β-tricalcium phosphate (TCP) (Superpore EX; HOYA Technosurgical, Tokyo, Japan) grafting. Two cylindrical bone plugs were transplanted to the recipient sites at the talus (Figure 3f).

 

**Figure 4:**
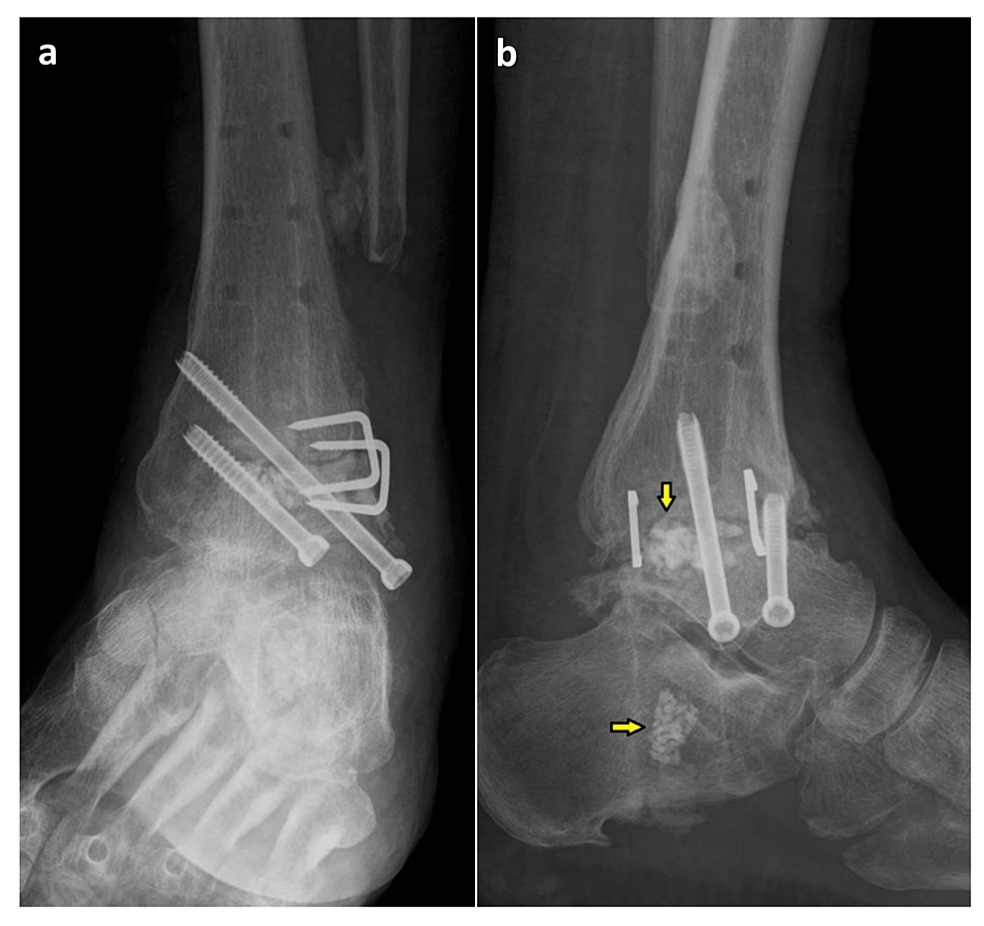
Plain radiographs just after surgery (a) anteroposterior and (b) lateral images

**Figure 5:**
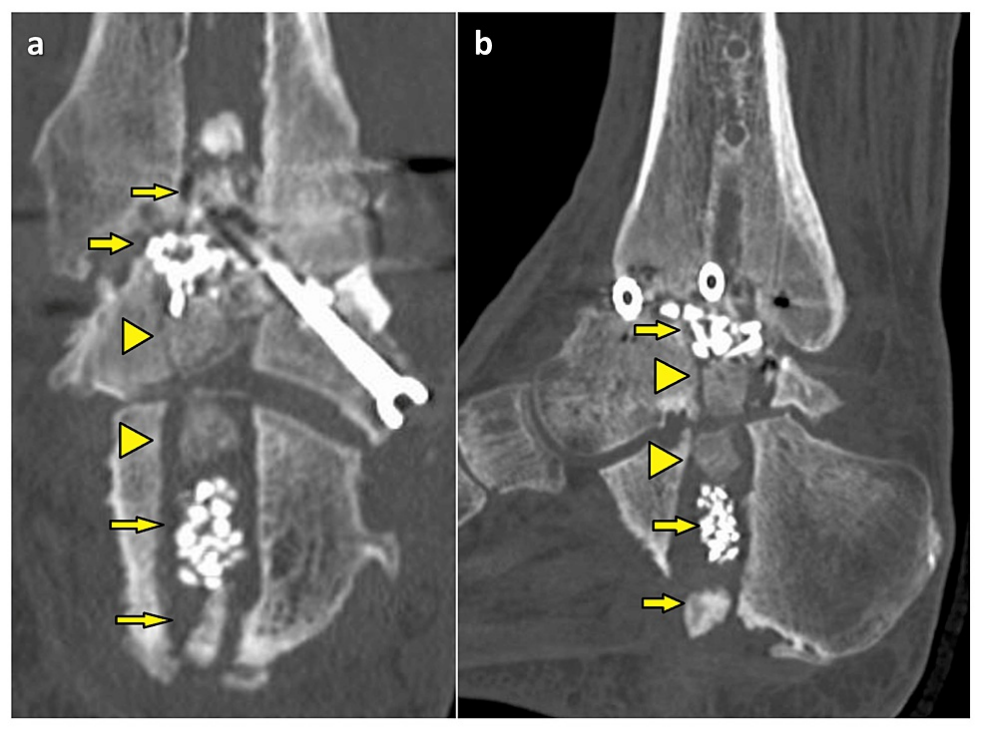
CT at one week after surgery (a) coronal and (b) sagittal images Autologous cancellous bone and artificial bone were filled in the ankle joint and intramedullary nail canal in the calcaneus (Arrows). Two osteochondral plugs (Arrowheads) were placed to reconstruct the subtalar joint. CT, computed tomography

The intramedullary canal of the calcaneus was filled with the β-TCP through a plantar incision (Figure 4, 5). 

**Figure 6:**
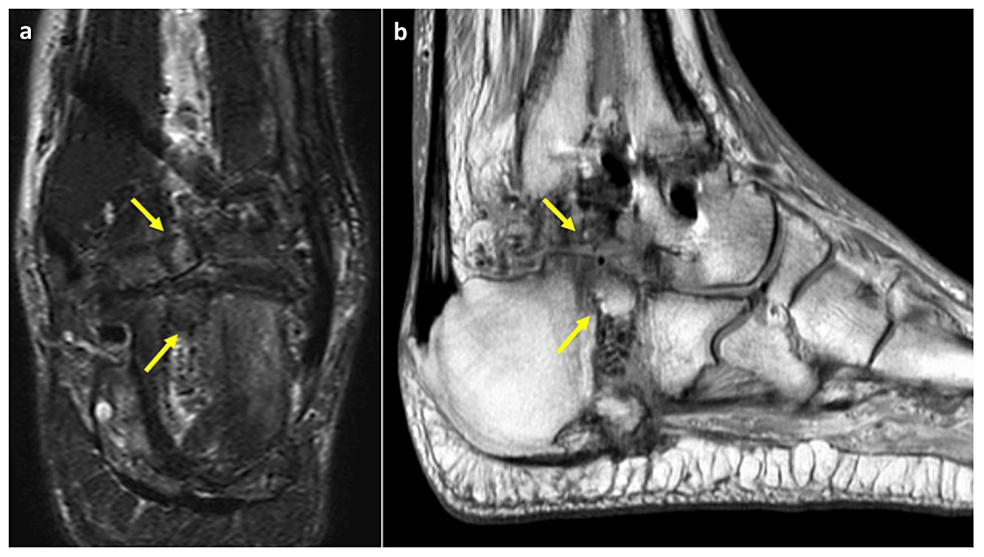
MRI at six months after surgery (a) coronal image of the T2-weighed fat suppression and (b) sagittal image of the proton density MRI, magnetic resonance imaging

Postoperatively, a short leg cast was applied for six weeks. Full weight bearing was permitted from four weeks using a short leg cast with a rubber walking heel. On radiographs taken two months after surgery, the bone union of the ankle joint was observed. Magnetic resonance imaging (MRI) six months after surgery revealed that the articular surface of the subtalar joint was flushed and osteochondral plugs were united with the surrounding bone (Figure 6).

**Figure 7:**
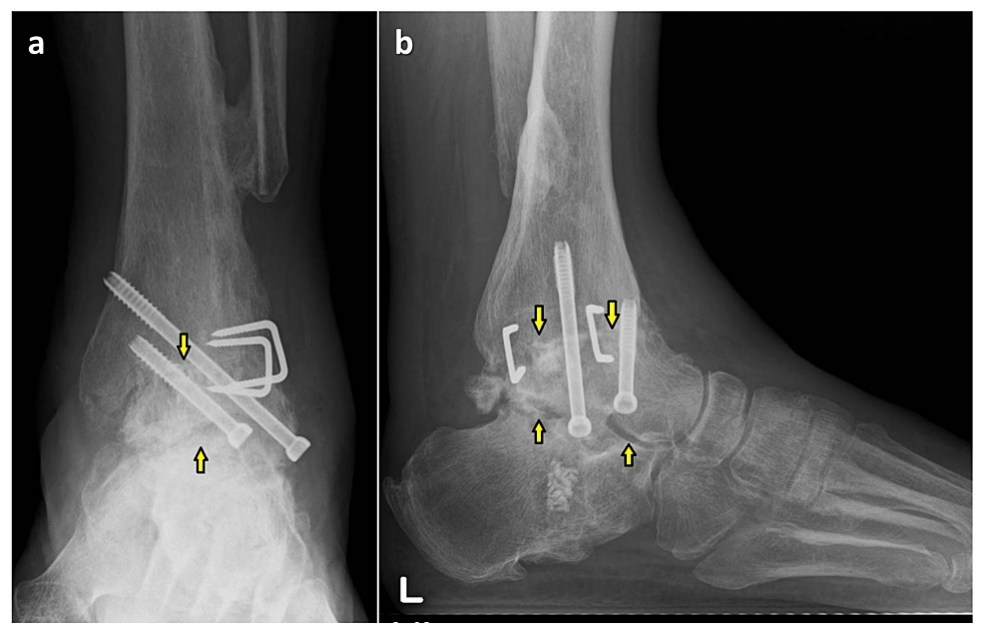
Plain radiograph at one year and three months after surgery (a) anteroposterior and (b) lateral images

As his plantar sensory disturbance was not alleviated, surgery was performed in an attempt to resolve this issue. The tibial nerve ruptured at the level of the medial malleolus, and a 5 cm gap was observed. Therefore, the deep peroneal nerve was transferred to the medial plantar nerve and the saphenous nerve to the lateral plantar nerve was performed. One year and three months postoperatively, there was no residual complaint of pain. The range of motion of midfoot was 5° in dorsiflexion and 10° in plantar flexion and 10° in supination and pronation of the calcaneus. The plain radiographs showed the bone union of the tibiotalar joint, and joint space of the subtalar joint was maintained (Figure 7).

The Japanese Society for Surgery of the Foot (JSSF) hindfoot scale improved from 53 to 84 points [10,11]. On the patient-reported SAFE-Q score, preoperative subscale scores improved after surgery, as follows: pain and pain-related questions, from 50 to 100 points; physical functioning and daily living, from 52.3 to 50 points; social functioning, from 45.8 to 58.3 points; shoe-related questions, from 41.7 to 66.7 points; general health and well-being, from 25 to 35 points [12,13].

## Discussion

Our case showed that salvage surgical reconstruction of the articular surface of the subtalar joint after the failure of tibiotalocalcaneal fusion with a retrograde intramedullary nail yielded good clinical outcomes. This technique is useful for revision cases of TTCA without subtalar joint preparation. A systematic review of the TTCA using a retrograde intramedullary nail revealed a 56% complication rate [4]. Among these, nonunion was a major complication. Kowalski et al. reported that nonunion of either the subtalar or tibiotalar joint was observed in 35.3% [14]. Gross et al. reported a union rate of 86% in the tibiotalar joint and 74% in the subtalar joint [15]. In previous reports, the fusion rate of TTCA using intramedullary nails was low. In our case, TTCA was performed using an intramedullary nail with fin-like longitudinal ridges. Instead of using distal transfixation screws, this intramedullary nail had four fins with sharp tips attached to the distal part of a cylindrical nail to stabilize the ankle and subtalar joints. Good clinical outcomes of arthrodesis for rheumatoid arthritis using this nail have been described [16,17]. Severe destructive arthrosis due to rheumatoid arthritis often affects both the ankle joint and subtalar joint. In these cases, the use of an intramedullary nail with fins is suitable because of the disappearance of the motion of the subtalar joint. On the other hand, the normal subtalar joint has a helical axis of 37.3±5.9° and a translation of 2.3±1.1 mm during the foot motion from extreme eversion to extreme inversion [18]. When an intramedullary nail with fins is used for ankle OA with a normal subtalar joint, subtalar motion may loosen the intramedullary nail, because the distal portion of the intramedullary nail is not transfixed with calcaneus. Loosening of the intramedullary nail at the calcaneus may induce the micromotion at the ankle joint. Subsequently, nonunion of the ankle joint and destruction of the subtalar joint occur. The indication of TTCA is severe rheumatoid arthritis, OA of tibiotalar and talocalcaneal joints, Charcot arthropathy, neuromuscular disease, trauma, severe deformity of clubfoot, congenital deformities, and pseudoarthrosis [19-21]. In addition, an asymptomatic subtalar joint is a contraindication for TTCA [22-24]. Isolated arthrodesis of the ankle joint should be performed in the case of the intact subtalar joint.

A previous report showed good clinical outcomes for osteochondral lesions of the talus by autologous osteochondral transplantation from the talar articular facet [25]. Good clinical outcomes for OLT after osteochondral autologous transplantation with graft harvested from a non-weight-bearing portion of the femoral condyle have been reported [26,27]. However, donor-site morbidity, such as discomfort and pain in the knee joint, with an incidence ranging from 0 to 54.5%, has been reported [28,29]. As the articular cartilage of the lateral aspect of the talus remained, osteochondral plugs did not need to be harvested from the knee joints in our case. Reconstruction of the articular surface of the subtalar joint using osteochondral autologous transplantation allowed the motion of the subtalar joint without pain, leading to an improvement of the subscale of pain and pain-related questions in the SAFE-Q. However, there is the possibility that degeneration subtalar joint would occur in the long-term follow-up because the quality of the articular cartilage of the osteochondral plugs from the remained articular cartilage in the talus was unclear. Reconstruction of the subtalar joint using OAT is a useful technique for failure cases of TTCA where the subtalar joint is preserved but there is partial destruction by an intramedullary nail canal. 

## Conclusions

We described a salvage surgery for nonunion of the tibiotalar and subtalar joints after the failure of tibiotalocalcaneal fusion using intramedullary nail fixation. Good clinical results were obtained by tibiotalar arthrodesis and reconstruction of the subtalar joint using OAT. OAT for the subtalar joint in combination with the tibiotalar arthrodesis could improve the symptoms, particularly pain, in the case of intramedullary nail failure without subtalar joint debridement.
